# Being uninformed on informed consent: a pilot survey of medical education faculty

**DOI:** 10.1186/1472-6920-5-12

**Published:** 2005-04-25

**Authors:** Brian E Mavis, Rebecca C Henry

**Affiliations:** 1 Office of Medical Education Research and Development, A202 East Fee Hall, Michigan State University, East Lansing, Michigan, 48824-1316, USA

## Abstract

**Background:**

This paper describes a pilot survey of faculty involved in medical education. The questionnaire focuses on their understanding of IRB policies at their institution, specifically in relation to the use of student assessment and curriculum evaluation information for scholarship.

**Methods:**

An anonymous survey was distributed to medical educators in a variety of venues. Two brief scenarios of typical student assessment or curriculum evaluation activities were presented and respondents were asked to indicate their likely course of action related to IRB approval. The questionnaire also asked respondents about their knowledge of institutional policies related to IRB approval.

**Results:**

A total of 121 completed surveys were obtained; 59 (50%) respondents identified themselves as from community-based medical schools. For the first scenario, 78 respondents (66%) would have contact with the IRB; this increased to 97 respondents (82%) for the second scenario. For both scenarios, contact with the IRB was less likely among respondents from research-intensive institutions. Sixty respondents (55%) were unsure if their institutions had policies addressing evaluation data used for scholarship. Fifty respondents (41%) indicated no prior discussions at their institutions regarding IRB requirements.

**Conclusion:**

Many faculty members are unaware of IRB policies at their medical schools related to the use of medical student information. To the extent that policies are in place, they are highly variable across schools suggesting little standardization in faculty understanding and/or institutional implementation. Principles to guide faculty decision-making are provided.

## Background

The Liaison Committee for Medical Education (LCME) expects medical schools to implement strategies for student assessment and curriculum evaluation that facilitate educational program management and improvement. In addition there is an expectation that faculty involved in these activities will reflect on what is learned and participate in the community of scholars to share this knowledge [1]. In practice, this has created confusion among faculty members involved in educational evaluation. Many faculty members are not clear under what circumstances institutional review board (IRB) approval is necessary. In some cases they conduct educational evaluations solely for institutional program improvement, while in other situations similar activities are undertaken to produce generalizable knowledge of interest to others [2,3]. As frequently occurs, student assessment and program evaluation activities intended for internal program monitoring or improvement yield outcomes that can lead to generalizable knowledge appropriate for scholarly publication, further blurring the distinction between evaluation and research [4]. A case in point were the recent allegations against the American Association of Medical Colleges, claiming that medical students completing the Graduation Questionnaire (GQ) were acting as subjects in a research study and human subjects protections were not followed. While many of the allegations were not upheld, the AAMC did agree to submit the GQ to an IRB for review [5].

There has been increasing debate among medical educators about the need to submit proposals for scholarship arising from student assessment and curriculum evaluation activities for IRB review. Here, the use of the word "scholarship" is used deliberately as it is not always synonymous with research, where there are clear criteria governing IRB review. The Common Rule defines research as "the systematic investigation, including research development, testing and evaluation, designed to develop generalizable knowledge [6]." Scholarship based upon quality improvement and program evaluation initiatives can fall into that uncertain territory that sometimes is equated with research while at other times is pursued for the single purpose of improving programs. Defining the point at which an internally directed program inquiry becomes publicly shared scholarship can vary across disciplines and/or the institution where the decision is rendered. Casarett and colleagues have published a more in depth discussion of criteria that might help distinguish research from other initiatives to document or improve program quality [7]. Our informal observation is that many medical educators set out to conduct evaluations to improve educational programs but after the evaluation is completed, the possibility of publishing or presenting the findings emerges, clouding the intent of the original activity. To add to the definitional murkiness, the determination of whether an initiative is research or some other activity is made locally by the institutional IRB, leading to variability across institutions.

In the context of evaluating educational programs, the question of human subjects protections for medical students is not new; it was posed over 20 years ago by Christakis [8]. Recent literature in this area suggests there is great variability in the extent to which medical schools address this issue [4]. Originally created to protect against abuses of human subjects in federally funded clinical research, 45 CFR 46 [9] is increasingly being applied more broadly to include research in the social sciences and education. Because medical education has historically used the curriculum and its related educational components as a laboratory for studying the teaching and learning process, ultimately with the goal of improving professional competency, the distinction between improvement and research becomes significant [4]. This tradition is consistent with Boyer [10], who urged educators to consider a broader view of scholarship, targeting the curriculum and the classroom as a source of inquiry. Faculty have been encouraged – if not mandated – to wear multiple hats [1,4], first as educators who participate in on-going teaching and evaluation, and second as scholars who uncover interesting observations and findings leading to more generalizable knowledge disseminated via professional meetings and publications. Given that accreditation and outcome assessment increasingly rely on learner performance data and that faculty are urged to publish from these efforts, the issue of using routine student assessment and program evaluation data for research remains salient. Until recently few medical education journals required evidence of IRB approval for manuscripts accepted for publication [4].

This study describes a pilot survey of faculty involved in medical education, and focuses on their understanding of IRB policies at their institutions related to the use of student information derived from assessment and evaluation activities for scholarship.

## Methods

We developed a brief questionnaire that described two short scenarios of typical student assessment or curriculum evaluation activities. For both cases, respondents were asked to choose from among several likely courses of action related to IRB approval: (a) submit an IRB application, (b) talk with the IRB chair, or (c) submit a conference abstract without IRB review.

### Case study 1

Your department has responsibility for the on-going evaluation of the clinical skills curriculum for preclinical medical students. In reviewing students' test scores from the course multiple-choice exams and faculty performance ratings of students, you identify some interesting relationships. In discussing these findings with the course director, you both agree that they have educational significance and decide to submit an abstract based on these data for the next regional medical education conference.

### Case study 2

Your department has responsibility for the on-going evaluation of the clinical skills curriculum for preclinical medical students. The clinical skills course coordinator inquired about the relationship between student performance in the first and second year of the clinical skills curriculum. Of particular interest is the bottom 20% of students based on faculty performance ratings. To answer this question, first and second year videotaped interviews for the bottom 20% of the class were reviewed by three faculty members, who rated the performance using standard checklists and rating scales. After reviewing the analyses of the data, the course director and faculty raters decided to submit an abstract for a regional medical education conference presentation.

The second section of the questionnaire asked respondents about their knowledge of institutional policies related to IRB approval. The final questionnaire item asked respondents to indicate if they were from a community-based medical school, research-intensive medical school or some other medical school/health professions program. This study was reviewed and approved by the Michigan State University Committee for Research Involving Human Subjects.

The anonymous survey was administered in person to 121 medical educators in a variety of different venues from Fall 2001 through Fall 2003 (Table 1). All of the fellowship programs included in this study focused on developing skills necessary for careers in medical education research.

**Table 1:** Recruitment venues for survey respondents

**Group**	**Date**	**Number**	**% of Total Sample**
Association of American Medical Colleges conference workshop	Nov. 2001	5	4%
Central Group on Educational Affairs conference session	March 2002	19	16%
Michigan State University primary care faculty development fellowship program	March 2002	19	16%
OBGYN faculty development fellowship	March 2002	20	17%
Surgery educational research fellowship	April 2002	9	7%
Michigan State University primary care faculty development fellowship program	Sept. 2002	19	16%
Surgery educational research fellowship	April 2003	11	9%
Michigan State University primary care faculty development fellowship program	Sept. 2003	19	16%

This was a descriptive study: the results were analyzed in terms of frequencies of responses for each of the questionnaire items. In addition, the open-ended responses were reviewed for themes, which were categorized for presentation in terms of percentages.

## Results

A total of 121 completed questionnaires were received. The exact response rate is unknown for the conference-based sessions due to incomplete records, but is greater than 90% for the fellowship sessions. Since not all parts of the questionnaire were completed, the specific sample sizes for each item are presented in the tables. The only identifier was the type of institution represented: 59 (50%) from community-based medical schools, 50 (42%) from research-intensive medical schools and 10 (8%) designated as another type of institution.

### Case studies

When presented with the first case study, respondents were fairly equally divided among the three courses of action provided; approximately two-thirds of the respondents would submit an IRB application or talk with the IRB chair (Table 2). For both cases, a small number of respondents indicated that they were unsure as to their likely course of action. Twenty-one respondents from research-intensive medical schools (43%) indicated that they would submit the abstract without involving the IRB compared to thirteen respondents (22%) from community-based medical schools *(Chi-Sq = 6.34, df = 3, p = 0.09, Odds Ratio = 2.60*).

**Table 2:** Responses to case study scenarios for research intensive and community-based medical schools

	Response to Case Scenario			
	Submit IRB application	Talk with IRB chair	Submit abstract without IRB review	Don't know

Case 1: χ^2 ^= *6.34*				
All respondents (N = 119)^1^	35 (29%)	43 (36%)	39 (32%)	2 (2%)
Research-intensive (N = 49)	14 (29%)	14 (29%)	21 (43%)	0 (0%)
Community-based (N = 58)	21 (36%)	22 (38%)	13 (22%)	2 (3%)
				
Case 2: χ^2 ^= 13.25 **				
All respondents (N = 118)^1^	56 (47%)	41 (35%)	19 (16%)	2 (2%)
Research-intensive (N = 49)	20 (41%)	14 (29%)	15 (31%)	0 (0%)
Community-based (N = 57)	30 (53%)	22 (39%)	3 (5%)	2 (4%)

1: Includes respondents who classified their institution as "other"

* p < .05; ** p < .01

For the second case study, ninety-seven (82%) respondents indicated involvement of the IRB. Though there was more IRB consultation overall for this case study, respondents from research intensive institutions more often reported (31% vs. 5%) that they would submit the abstract without IRB consultation than subjects from community-based schools *(Chi-Sq = 13.25, df = 3, p = 0.004, OR = 7.94).*

### Knowledge about institutional policies

Sixty-one respondents (52%) indicated that their IRB was university-based, compared to forty-six (39%) who interact with IRBs through their medical center (Table 3). This dichotomy is important insofar as it distinguishes between a centralized university IRB addressing all human subjects concerns and institutions where there are multiple specialized IRBs. Many of the applications to university-based IRBs focus on the protection of students as human subjects, and IRB members are likely to have more experience with educational research protocols. Historically, IRBs within medical centers have focused on the protection of patients as human subjects, both as clinical research participants and more recently with the introduction of quality improvement initiatives.

**Table 3:** Knowledge of institutional policies for research intensive and community-based medical schools

	Respondent Group			Test Statistic
	All Respondents^1^	Research Intensive	Community-Based	

For medical education studies that require IRB review, to which IRB would you submit your application? (N = 117)				
University	61 52%	29 58%	27 47%	χ^2 ^= 3.66
Medical center	46 39%	20 40%	24 42%	
Other	9 8%	1 2%	5 9%	
Don't know	1 1%	0 0%	1 2%	
				
Does your institution have stated policies on the use of existing educational evaluation data for faculty scholarship? (N = 110)				
Yes	30 27%	7 16%	11 20%	χ^2 ^= 1.71
No	20 18%	15 33%	12 22%	
Unsure	60 55%	23 51%	32 58%	
				
Which best describes procedures in place at your medical school for obtaining consent from students to use their performance data and test scores for educational research & scholarship? (N = 115)				
Students can decline consent	24 21%	6 12%	3 5%	χ^2 ^= 2.75
Matriculation conditional on consent	11 10%	8 16%	14 25%	
There are no procedures	50 43%	21 43%	26 46%	
Don't know	30 26%	14 29%	13 23%	
				
Have you participated in discussions with others at your institution about IRB requirements for using evaluation data for faculty scholarship? (N = 119)				
Faculty in your department	53 45%	23 46%	24 41%	χ^2 ^= 0.31
Faculty in other departments	24 20%	13 26%	11 19%	χ^2 ^= 0.85
College faculty meetings	9 8%	6 12%	0 0%	χ^2 ^= 7.49**
Dean, administrator, etc.	19 16%	12 24%	5 9%	χ^2 ^= 4.96*
Other	17 14%	6 12%	9 15%	χ^2 ^= 0.24
No discussions reported	50 41%	21 42%	25 42%	χ^2 ^= 0.002

1: Includes respondents who classified their institution as "other"

* p < .05; ** p < .01

Sixty respondents (55%) were unsure if their institutions had policies in place addressing the use of educational evaluation data for scholarly dissemination. Thirty respondents (27%) indicated that their institutions did have a policy in place. Only eleven respondents (10%) reported that their institution made matriculation contingent on students providing consent to have their academic information used for faculty research. Twenty-four respondents (21%) indicated that students can decline to give consent for participation in faculty scholarship. There were no differences in the responses for participants from research-intensive institutions compared to community-based medical schools.

Twenty-four of the participants (20%) provided written comments elaborating their responses about institutional policies; some respondents made multiple comments so the number of comments exceeds the number of respondents. Six of this subgroup (30%) reported simply that an IRB application was required, while some indicated the specific level of review as exempt (N = 7; 35%) or expedited (N = 4; 20%). Three respondents (15%) specified that the proposal was exempt only if the data were anonymous, and one respondent (5%) indicated that students must provide consent. One respondent (5%) wrote that there was no explicit policy but faculty were advised to consult with the IRB chair, while two respondents (10%) distinguished between evaluation for program improvement versus generalizable knowledge leading to scholarship. Another faculty member (5%) replied that secretarial staff took care of matters related to the IRB. Finally, one respondent (5%) confessed that he or she had never heard of this issue prior to involvement in the fellowship program.

### Discussions with colleagues

Respondents were asked if they had participated in discussions with others at their institutions related to IRB approval for the use of student evaluation data for faculty scholarship. A significant proportion of respondents (N = 50, 41%) did not report any prior discussions with others at their institution regarding IRB requirements; only one respondent (0.8%) reported participating in discussions with all five of the groups listed. Overall, when faculty reported engaging in such discussions, it was most likely to have occurred with faculty members within their own (N = 53, 45%) or other (N = 24, 20%) departments. This was consistent across respondent subgroups. More respondents from research intensive institutions reported discussions at college level faculty meetings *(Chi-Sq = 7.49, df = 1, p = 0.006) *and with institutional administrators *(Chi-Sq = 4.96, df = 1, p = 0.03, OR = 3.41) *than respondents from community-based medical schools.

## Discussion

There has been increasing interest among medical educators about the need for human subjects protections for faculty scholarship derived from student assessment and curriculum evaluations activities. Tomkowiak and Gunderson [1] recently mused how many medical educators were aware that scholarship derived from the evaluation of a curricular innovation could be considered research subject to federal human research standards and governance? The results of this pilot survey suggest that many faculty members are unaware of relevant policies at their home institutions; to the extent that policies for human subjects review and approval are in place, faculty understanding and reporting of these policies are highly variable across institutions. The implication is that many faculty active in educational research and evaluation lack an understanding of their institutions policies regarding the use of students or other learners as research subjects. In rare cases, faculty delegate this responsibility to staff members. The variability with which institutions have addressed this issue, or how faculty have acted on these concerns, makes discourse about the human subjects concerns difficult for those involved in medical education as a profession. It suggests a lack of standards and standardization with respect to IRB review of educational research of a magnitude less common in other fields of scientific inquiry.

In practice, IRB standards are subject to local interpretation when institutional procedures are established. The data from the two case studies presented suggest that while a majority of faculty members would minimally seek IRB consultation for educational research, respondents from research-intensive institutions have been less likely to involve the IRB. Respondents from research-intensive institutions were more likely to engage in discussions about this issue. This could mean that there is a better shared understanding of the policies and practices at the specific institution that might not require application for IRB review for educational research. Conversely, it could signal a continuing legacy of research practices among faculty that is independent of institutional policy as well as Office of Human Research Protections (OHRP) guidelines, which indicate that the IRB not the investigator determines whether a study qualifies for exempt or expedited review [11]. Additional information distinguishing between exempt, expedited and full review procedures can be found at the OHRP website [11]. In some cases, respondents reported that they would contact the IRB chair, presumably for consultation. This demonstrates critical information seeking behavior and such consultation would likely yield a recommendation from the IRB chair about the need to apply for IRB review. Nonetheless, consultation with the IRB chair cannot be construed as equivalent to seeking IRB review since the faculty member's ultimate course of action is unknown. These findings highlight the importance of institutional culture in shaping faculty practices related to research and scholarship.

This study focuses on educational research and evaluation with students as human subjects. Much of the information used in such investigations, such as grades, test scores and performance ratings, are natural byproducts of students in their role as learners. These investigations appear to be minimal risk and are typically considered exempt by IRBs when the information is low-risk, presented in aggregate and used by faculty members responsible for the specific curricular component. However, the complexity of educating medical students presents many opportunities for collecting a wide range of personal information, linking student information across datasets, studying subgroups of students and in some instances, providing faculty with access to student information to which they might not otherwise have access. It is likely that students are not aware of the extent to which this occurs within their institution or the circumstances and protections surrounding such occurrences. In situations such as these, IRB review can weigh the risks and benefits to assure adequate protections for students. Some might argue that IRB review is necessary even when the information is used for internal program monitoring and quality control [7]. Involvement of the IRB in educational evaluations does not have an impact on students' obligation to complete the requirements of their curriculum, but can have an impact on whether or not this information can be used for other purposes such as conference presentations or publications.

This study was designed as an exploratory descriptive investigation, and as such is limited as a pilot sample based on a small number of respondents. However we have attempted to sample across a variety of cohorts of faculty actively involved in medical education. Compared with other medical school faculty one could hypothesize that they should be better informed about IRB policies as their professional emphasis is in medical education and the products of their work would likely involve students as subjects. Faculty have the right and responsibility to collect information, including student assessments, to improve instruction, curricula and educational outcomes. However, this right does not extend to the use of this information for publication or scholarship without student consent or IRB waiver of consent.

Because the questionnaire was anonymous little is known about the respondents in terms of their academic background and training or experience in medical education. In addition, the findings are derived for faculty reports of institutional policy rather than a review of actual institutional policies. However, even the preliminary comparisons of individuals from different types of institutions show significant differences suggestive of variability within the profession. A more systematic sample of subjects and a questionnaire that includes more respondent information is needed to provide a more comprehensive perspective faculty knowledge and practice related to human subjects concerns for educational evaluation and research.

## Conclusion

Many faculty members lack understanding about their institutions policies for IRB review of educational evaluation and other quality improvement strategies. Professional organizations and journals in medical education could assist by developing position statements and clear expectations about protecting students when their performance and survey data are used in publications and presentations.

Given this inconsistent understanding of institutional policies, what might assist in educating faculty about when to seek IRB review? Some have suggested a series of questions that might guide us on when educational research may be exempt [12]. Central to these questions is:

1. When conducting an evaluation, faculty should consider whether or not the activity is research (is it designed to contribute to generalizable knowledge?).

2. If the activity is research, do the learners meet the criteria for human subjects?

3. If the activity is human subject research, the faculty member should seek IRB consultation regarding whether or not the research activity is exempt.

4. If research was not an original intent of the evaluation, but the faculty member later determines that the results of the evaluation might contribute to generalizable knowledge, the above principles become applicable.

For any evaluation the Office of Human Research Protections (OHRP) recommends that the local IRB or another independent institutional authority be consulted in making these determinations [11].

Perhaps a more fundamental concern is for those faculty members who never seek IRB review or consultation in the first place. Our advice to all faculty involved in activities that could be construed as medical education research is to assume that any medical students or others involved in the educational process, such as residents, faculty or standardized patients, might well meet the criteria to be considered human subjects and appropriate consultation should be sought.

## Competing interests

The author(s) declare that they have no competing interests.

## Authors' contributions

BM contributed to the design of the study, analysis of the data and preparation of the first draft of the manuscript. RH contributed to the design of the study, data collection and revisions to the draft manuscript.

## Pre-publication history

The pre-publication history for this paper can be accessed here:


